# Tumour-draining axillary lymph nodes in patients with large and locally advanced breast cancers undergoing neoadjuvant chemotherapy (NAC): the crucial contribution of immune cells (effector, regulatory) and cytokines (Th1, Th2) to immune-mediated tumour cell death induced by NAC

**DOI:** 10.1186/s12885-018-4044-z

**Published:** 2018-02-02

**Authors:** Viriya Kaewkangsadan, Chandan Verma, Jennifer M. Eremin, Gerard Cowley, Mohammad Ilyas, Oleg Eremin

**Affiliations:** 1 Division of Gastrointestinal Surgery, Nottingham Digestive Diseases Centre, Faculty of Medicine and Health Sciences, University of Nottingham, E Floor West Block, Queen’s Medical Centre, Derby Rd, Nottingham, NG7 2UH UK; 2 Research & Development Department, Lincoln Breast Unit, Lincoln County Hospital, Greetwell Road, Lincoln, LN2 5QY UK; 3 Department of Pathology, PathLinks, Lincoln County Hospital, Greetwell Road, Lincoln, LN2 5QY UK; 4 Academic Department of Pathology, Faculty of Medicine and Health Sciences, University of Nottingham, A Floor West Block, Queens Medical Centre, Derby Road, Nottingham, NG7 2UH UK; 5 Department of Surgery, Phramongkutklao Hospital and College of Medicine, 315 Rajavithi Road, Bangkok, 10400 Thailand

**Keywords:** Axillary lymph node, Breast cancer, Neoadjuvant chemotherapy, Tumour microenvironment, Tumour-infiltrating lymphocyte subsets, Cytokines

## Abstract

**Background:**

The tumour microenvironment consists of malignant cells, stroma and immune cells. In women with large and locally advanced breast cancers (LLABCs) undergoing neoadjuvant chemotherapy (NAC), tumour-infiltrating lymphocytes (TILs), various subsets (effector, regulatory) and cytokines in the primary tumour play a key role in the induction of tumour cell death and a pathological complete response (pCR) with NAC. Their contribution to a pCR in nodal metastases, however, is poorly studied and was investigated.

**Methods:**

Axillary lymph nodes (ALNs) (24 with and 9 without metastases) from women with LLABCs undergoing NAC were immunohistochemically assessed for TILs, T effector and regulatory cell subsets, NK cells and cytokine expression using labelled antibodies, employing established semi-quantitative methods. IBM SPSS statistical package (21v) was used. Non-parametric (paired and unpaired) statistical analyses were performed. Univariate and multivariate regression analyses were carried out to establish the prediction of a pCR and Spearman’s Correlation Coefficient was used to determine the correlation of immune cell infiltrates in ALN metastatic and primary breast tumours.

**Results:**

In ALN metastases high levels of TILs, CD4^+^ and CD8^+^ T and CD56^+^ NK cells were significantly associated with pCRs.. Significantly higher levels of Tregs (FOXP3^+^, CTLA-4^+^) and CD56^+^ NK cells were documented in ALN metastases than in the corresponding primary breast tumours. CD8^+^ T and CD56^+^ NK cells showed a positive correlation between metastatic and primary tumours. A high % CD8^+^ and low % FOXP3^+^ T cells and high CD8^+^: FOXP3^+^ ratio in metastatic ALNs (tumour-free para-cortex) were associated with pCRs. Metastatic ALNs expressed high IL-10, low IL-2 and IFN-ϒ.

**Conclusions:**

Our study has provided new data characterising the possible contribution of T effector and regulatory cells and NK cells and T helper1 and 2 cytokines to tumour cell death associated with NAC in ALNs.

**Trial registration:**

The Trial was retrospectively registered. Study Registration Number is ISRCTN00407556.

**Electronic supplementary material:**

The online version of this article (10.1186/s12885-018-4044-z) contains supplementary material, which is available to authorized users.

## Background

There is increasing evidence that anti-cancer immune mechanisms play an important role in the induction, development and dissemination of malignant disease in man [1–4]. Both innate and adaptive immune cells have been documented in a wide range of human solid cancers (breast, gastrointestinal, urogenital, head and neck and melanoma) and the presence of a prominent lymphocytic infiltrate is associated with a good long-term clinical outcome [5–8, 4]. In women with breast cancer undergoing neoadjuvant chemotherapy (NAC) a prominent presence of tumour-infiltrating lymphocytes (TILs), has been shown to be associated with an increased incidence of a complete pathological response (pCR) (a recognised surrogate marker of improved clinical outcome) in the primary breast tumour [9–13]. The presence of TILs infiltrating tumour deposits in tumour-draining axillary lymph nodes (ALNs) and the contribution to immune-mediated tumour cell death and pCR, however, is less well understood and poorly studied.

Although most chemotherapeutic drugs produce short-lived inhibitory effects on innate and adaptive immune cells, some (anthracyclines, taxanes, cyclophosphamide, capecitabine and gemcitabine) can modulate (enhance or suppress) specific aspects of immune mechanisms and activate immune-mediated tumour cell death contributing to the good pathological responses documented in the primary cancers [14–20, 13].

We and others had previously documented the presence of different lymphocyte subsets (T effector cells [CD4^+^, CD8^+^], T regulatory cells [Tregs: FOXP3^+^, CTLA-4^+^], natural killer cells [NK: CD56^+^]) infiltrating breast tumours in women with large and locally advanced breast cancers (LLABCs), and showing a significant association (except for FOXP3^+^ T cells) with a good pathological response, in particular to a pCR, following NAC [21–26, 13]. A pCR in the breast is recognised as a surrogate marker of a good long-term clinical outcome [27, 28]. A pCR, however, is more frequent in high grade and triple negative breast tumours [28].

In breast cancer, metastatic tumour spread to ALNs carries a poor prognosis and is one of the strongest predictors of a poor long-term survival [29, 30]. A more reliable surrogate marker of clinical outcome is a pCR in tumour-draining metastatic ALNs, even in the absence of an optimal pathological response in the primary tumour in the breast [28]. The relevance and prognostic significance of TILs and different lymphocyte subsets (effector, regulatory) in the ALN metastatic deposits, however, is less well studied [31–34]. The contribution of TILs effector and regulatory lymphocyte subsets to tumour cell death with NAC is even less well studied and documented.

We wished to establish whether these key lymphocyte subsets circulating in blood and infiltrating the primary cancer in women with LLABCs, that we had previously shown to possibly play an important role in inducing immune-mediated tumour-cell death during NAC, contributed to the pCR in ALN metastatic deposits, thereby enhancing long-term survival. We also wished to document which suppressive factors (cellular, humoral) may have contributed to a failure to achieve a pCR in metastatic ALNs.

## Methods

### Patients and samples

Studies were carried out on paraffin-embedded tumour-draining ALN specimens from 33 women with LLABCs (> 3 cm, T3-4, N0-2, M0). The breast tumour specimens had been used in a previous study to investigate primary tumour infiltration by immune cells [13]. Twenty four patients had nodal metastases, 9 patients were without nodal metastases; 20 out of 24 patients with nodal metastases (confirmed in post-surgical resection specimens) had additional pre-NAC core-needle biopsy samples of metastatic tumours in ALNs. The specimens were from patients enrolled in a study of NAC between 2008 and 2011 [28]. The NAC trial evaluated the effect of the addition of capecitabine (X) to docetaxel (T) preceded by adriamycin and cyclophosphamide (AC). The clinical status of ALNs was assessed by clinical examination and high–resolution ultrasonography. Patients with clinically negative nodal status did not undergo pre-NAC ALN biopsies. Patients with clinically positive nodal status underwent pre-NAC ultrasound-guided core biopsies. Fine needle aspiration cytology was not carried out. Pathological responses were assessed from the surgical resection specimens following completion of NAC. Established and previously published grading criteria were used to define histopathological responses in the breast [35, 36]. Pathological responses in metastatic tumours in ALNs were defined as pCR (grade 3: complete disappearance of tumour deposits or replacement by fibrosis in a previously histologically confirmed metastatic ALN); pathological partial response (grade 2: residual metastatic tumour deposits present with evidence of tumour destruction and replacement by fibrosis); no pathological response (grade 1: metastatic tumour deposits remain with no evidence of fibrosis). Histopathological sections of pre-NAC ultrasound-guided core-cut biopsies of breast tumours and ALNs were assessed. Histopathological sections of post-NAC (surgical specimens) of breast tumours and ALNs were graded by an experienced breast pathologist. The histopathological findings were discussed at a Multidisciplinary Meeting and a consensus decision made. The type and level of immune cell infiltration in primary breast tumours of corresponding patients were used to compare and correlate with the type and level of immune cell infiltration in metastatic tumours in ALNs. The data from the primary tumours was obtained from our previous study [13] (Additional files 1 and 2).

The study was given approval by the Leicestershire, Northamptonshire & Rutland Research Ethics Committee 1: Reference Number 07/H0406/260; Favourable Opinion 24/01/2008. All patients enrolled in the study gave informed consent to participate in and to publish the results of the study. The study Registration is ISRCTN00407556.

### Immuno-histochemical assessment

Immuno-histochemical (IHC) assessments of immune cell subsets and expression of cytokines and biological molecules were performed in 4-μm tissue sections. Briefly, paraffin-embedded tissue sections were dewaxed and rehydrated using xylene and graded alcohol. Citrate buffer, pH 6.0, at 98 °C was added for 20 min (mins) for antigen retrieval. After serial blocking, the sections were incubated with the primary monoclonal antibody (MAb) against CD4 (Dako, M7310, clone 4B12), 1:80 dilution for 30 mins at room temperature (RT); MAb against CD8 (Dako, M7103, clone C8/144B), 1:100 dilution for 30 mins at RT; MAb against FOXP3 (Abcam, ab20034, clone 236A/E7), 20 μg/ml for 30 mins at RT; MAb against CTLA-4 (Santa Cruz Bio, sc-376,016, clone F-8), 1:300 dilution for 30 mins at RT; MAb against PD-1 (Abcam, ab52587, clone NAT105), 1:100 dilution for 30 mins at RT; MAbs to CD56 (Dako, M7304) at a 1:50 dilution for 30 mins at RT; MAb against interleukin-1 (IL-1) (Abcam, ab8320, clone 11E5), 1:150 dilution overnight at 4 °C; MAb against IL-2 (Abcam, ab92381, clone EPR2780), 1:500 dilution for 30 mins at RT; polyclonal Ab against IL-4 (Abcam, ab9622), 4 μg/ml for 30 mins at RT; polyclonal Ab against IL-10 (Abcam, ab34843), 1:400 dilution for 30 mins at RT; polyclonal Ab against IL-17 (Abcam, ab9565), 1:100 dilution for 30 mins at RT; polyclonal Ab against interferon-gamma (IFN-γ) (Abcam, ab9657), 4 μg/ml for 30 mins at RT; MAb against transforming growth factor-beta 1 (TGF-β1) (Abcam, ab64715, clone 2Ar2), 12 μg/ml overnight at 4 °C; polyclonal Ab against PD-L1 (Abcam, ab58810), 2.5 μg/ml for 15 mins at RT; MAbs to indole-amine 2, 3-dioxygenase (IDO) (Abcam, ab55305) at a concentration of 0.75 μg/ml for 15 mins at RT; MAbs to vascular endothelial growth factor (VEGF) (Dako, M7273) at a 1:50 dilution for 30 mins at RT. The Novolink™ polymer detection system, Leica RE7280-K with polymeric horseradish peroxidase (HRP)-linker antibody conjugates and di-amino-benzidine (DAB) chromogen, was used for enzyme-substrate labelling. Finally, the sections were counterstained with haematoxylin, dehydrated and mounted in DPX mounting medium. Positive and negative staining controls were carried out with tonsil sections except for CTLA-4 (colon carcinoma sections), IL-1, IL-4 and TGF-β (kidney carcinoma sections), IL-10 and IDO (normal colon sections). Negative staining controls were demonstrated by omitting the primary antibody. Positive and negative staining were simultaneously performed with every IHC staining run.

### Semi-quantification of IHC sections

Whole tissue sections were studied rather than microarrays in order to minimise sampling bias. Representative examples of high power fields (HPFs: 400× magnification) are shown for clarity and ease of presentation of the Figures. All sections were scored without knowledge of the patients’ clinical and pathological parameters.

To evaluate TILs in haematoxylin and eosin (H&E)-stained sections, TILs were reported as the % of the metastatic tumour epithelial nests that contained infiltrating lymphocytes. Scores of > 60% were considered to be high levels of infiltration, while ≤60% were considered to be low levels of infiltration [9, 12, 37].

To evaluate the presence and extent of specific T cell and NK cell subsets in the metastatic tumours, the average numbers of brown membrane/nuclear-stained cells, regardless of the intensity, in contact with metastatic tumour cells or within the metastatic tumour cell nests, were counted in 5 HPFs [22, 38].

To evaluate the presence and extent of specific T cell subsets (CD4^+^, CD8^+^, FOXP3^+^) in the ALNs, the positively-stained cells were quantified as the average % of all cells per HPF in non-tumour involved para-cortical areas of ALNs. The average number of cell counts per HPF with the greatest accumulations of positively-stained less prominent cell populations (CD56^+^, PD1^+^, CTLA-4^+^), established by prior scanning at low magnification, was carried out [33, 39].

To evaluate the expression of cytokines and biological molecules in ALNs, the presence of IL-1, IL-2, IL-4, IL-10, IL-17, IFN-γ, TGF-β, IDO, VEGF and PD-L1 was assessed in whole tissue sections of non-metastatic, para-cortical areas and semi-quantified by using the H scoring system. The H score was calculated by multiplying the % of positive cells by a factor representing the intensity of immune-reactivity (1 for weak, 2 for moderate and 3 for strong), giving a maximum score of 300. The staining grade of intensity was defined according to the majority of the DAB staining intensity throughout a specimen. A score of < 50 was considered negative and a score of 50-100 was considered weakly positive (1+). A score of 101-200 was regarded as moderately positive (2+) and a score of 201-300 as strongly positive (3+). Negative and 1+ were considered as low expression whereas 2+ and 3+ were considered as high expression.

### Statistical analysis

Statistical analyses were performed with the IBM SPSS statistics software, version 21 (SPSS Inc., Chicago, IL, USA). Where the data did not follow a normal distribution, non-parametric tests (Mann-Whitney U test [between two variables/groups]) were used to compare the groups based on pathological responses (pCR and non pCR) and clinical-pathological parameters. Pearson Chi-Square test was performed to compare the binomial data (negative/low versus high) on expression of cytokines/biological molecules between groups. To evaluate and compare the related-sample data between metastatic tumours and corresponding primary tumours, the Related-Samples Wilcoxon Signed Rank test and Related-Samples McNemar test were performed for comparing the number of cell counts (continuous data) and the level of TILs (binomial data), respectively. The correlations of immune cell infiltrations between metastatic tumours in ALNs and primary tumours in breast were carried out using the Spearman’s Correlation Coefficient (rho). A probability value (*p* value) of equal to or less than 0.05 (2-tailed) was considered statistically significant. Based on our previous findings with Tregs and using the N Query Advisor 6.0 analysis software, we established that the minimum number of patients (*n* = 7) in a sample group relating to the pathological response groups was appropriate. However, the study possesses several assays of different parameters, the sample size of at least 7 in each group may not be appropriate for some of the tests.

## Results

### High levels of intra-tumoural TILs in ALN metastases were significantly associated with a PCR in the tumour-involved ALNs (*n* = 20) following NAC

**Fig. 1:**
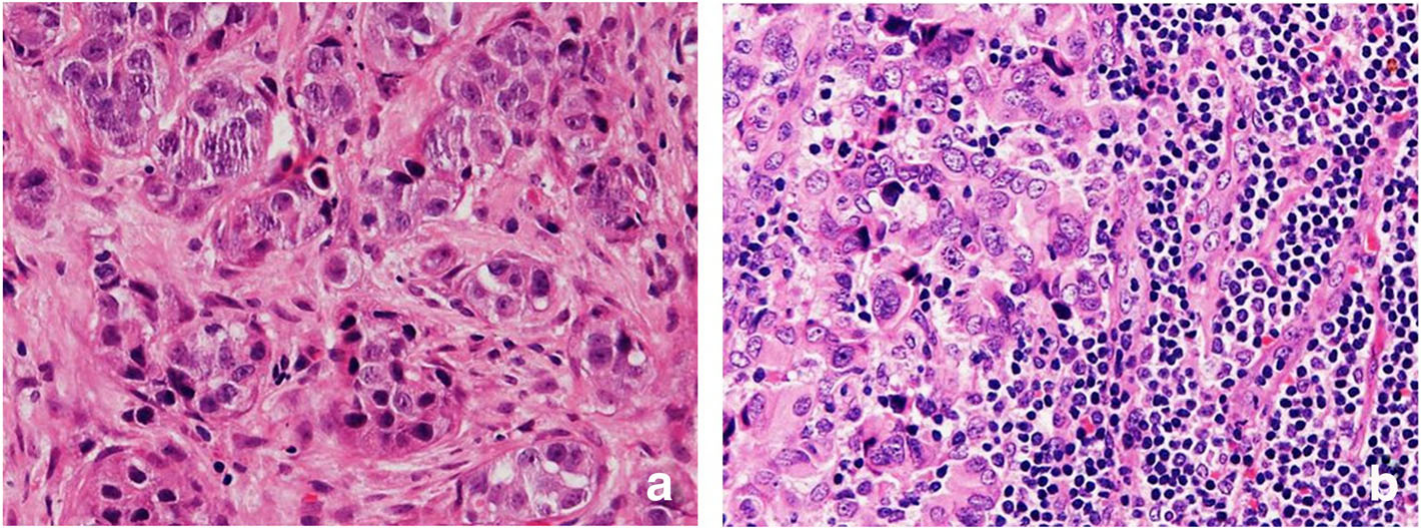
TILs in the sections of metastatic tumours, using H&E staining, at 400× magnification. **a**: low level of lymphocytic infiltration; **b**: high level of lymphocytic infiltration. Low level of TILs defined as ≤60% of tumour nests infiltrated by lymphocytes. High level of TILs defined as > 60% of tumour

The levels of TILs present in tumour cell nests in metastatic ALNs were assessed in pre-NAC lymph node biopsies (n = 20). Nine patients had pCR in metastatic tumour deposits in their ALNs. Eight of these 9 patients had concordant pCRs in the primary breast tumours. High levels of TIL infiltration (> 60% of metastatic tumour cell nests containing lymphocytes) was found in 55.6% (5 out of 9) of metastatic ALNs which subsequently had a pCR. In contrast, low levels of TILs were associated with only 9.1% (1 out of 11) of metastatic ALNS showing a pCR after NAC (*p* = 0.024) (Table 1) (Fig. 1: a, b).

**Table 1:** High Levels of Tumour-infiltrating Lymphocytes (TILs) in Pre-NAC^(a)^ ALN^(b)^ Metastatic Tumours: Association with a PCR Following NAC

	Groups	Pre-NAC			
		Low Infiltration (n)	High Infiltration (n)	Pearson Chi-Square Value (PCR Versus Non PCR)	*P* Value
TILs (*n* = 20)	Pathological Complete Response (PCR, *n* = 9)	4	5	5.089	0.024^c^
	Non Pathological Complete Response (Non PCR, *n* = 11)	10	1		

^(a)^NAC: Neoadjuvant chemotherapy; ^(b)^ ALN; Axillary lymph nodes; ^c^ Statistically significant

### High levels of intra-tumoural T Effector cell subsets (CD4^+^, CD8^+^) and CD56^+^ NK cells in ALN metastases were significantly associated with a PCR in tumour-involved ALNs (*n* = 20) following NAC

**Fig. 2:**
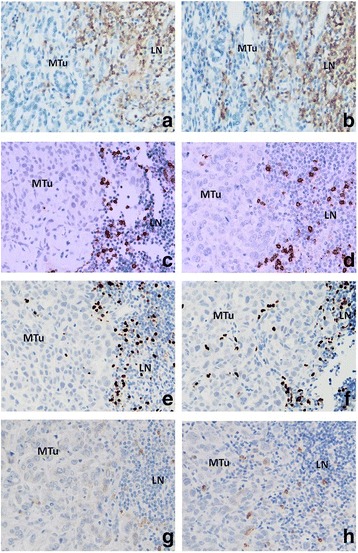
CD4^+^ (**a**, **b**), CD8^+^ (C, D) T lymphocytes, FOXP3^+^ Tregs (**e**, **f**) and CD56^+^ NK cells (**G**, **H**) in the sections of metastatic tumours, using IHC staining, at 400× magnification. Briefly, heat-mediated antigen retrieval was performed using citrate buffer, pH 6 (20 mins). The sections were then incubated with MAbs to CD4 (Dako, M7310) at a 1:80 dilution for 30 mins at RT, MAbs to CD8 (Dako, M7103) at a 1:100 dilution for 30 mins at RT, MAbs to FOXP3 (Abcam, ab20034) at a concentration of 20 μg/ml for 30 mins at RT, MAbs to CD56 (Dako, M7304) at a 1:50 dilution for 30 mins at RT. Polymeric HRP-linker antibody conjugate was used as secondary antibody. DAB chromogen was used to visualize the staining. The sections were counterstained with haematoxylin. **a**, **c**, **e**, **g** low level of CD4^+^, CD8^+^ T cell, FOXP3^+^ Treg, CD56^+^ NK cell infiltration respectively; **b**, **d**, **f**, **h**: high level of CD4^+^, CD8^+^ T cell, FOXP3^+^ Treg, CD56^+^ NK infiltration respectively. The average number of brown membrane-stained cells (CD4^+^, CD8^+^ T cells, CD56^+^ NK cells) and brown nuclear-stained cells (FOXP3^+^ Tregs) regardless of intensity, in contact with tumour cells or within tumour cell nests per HPF was counted. MTu: Metastatic tumour nest; LN: Lymphoid tissue

The levels of lymphocyte subsets infiltrating metastatic tumour cell nests in ALNs were assessed in pre-NAC lymph node biopsies (n = 20) (pre-NAC ultrasound-guided core biopsies from patients with clinically positive nodal status). High levels of infiltration (> 60% of metastatic tumour cell nests containing lymphocytes) by CD4^+^ and CD8^+^ T cells was significantly associated with a pCR (*p* = 0.004 and *p* = 0.001, respectively) following NAC (Table 2) (Fig. 2: a, b; c, d). Infiltration by high levels of CD56^+^ NK cells was also significantly associated with a pCR in the metastatic ALNs (*p* = 0.010) (Fig. 2: g, h). There was, however, no significant association between the level of FOXP3^+^ and CTLA-4^+^ T cells and a pCR in metastatic ALNs following NAC (Fig. 2: e, f).

**Table 2:** High Levels of T Effector (CD4^+^, CD8^+^) and CD56^+^ NK Cells in Pre-NAC ^(a)^ ALN^(b)^ Metastatic Tumours: Association with a PCR Following NAC

Lymphocyte Subsets (n = 20)	Groups	Tumour Infiltration Median (range)^(c)^	P Value^(d)^(PCR Versus Non PCR)
CD4^+^	Pathological Complete Response (PCR, *n* = 9)	65.0 (19.4-157.4)	0.004^e^
	Non Pathological Complete Response (Non PCR, *n* = 11)	13.2 (0.6-100.8)	
CD8^+^	Pathological Complete Response (PCR, *n* = 9)	99.2 (33.2-160.8)	0.001^e^
	Non Pathological Complete Response (Non PCR, *n* = 11)	11.6 (0.4-93.0)	
FOXP3^+^	Pathological Complete Response (PCR, *n* = 9)	18.0 (5.0-73.6)	0.152
	Non Pathological Complete Response (Non PCR, *n* = 11)	6.4 (1.0-20.4)	
CTLA-4^+^	Pathological Complete Response (PCR, *n* = 9)	2.6 (0.4-11.6)	0.112
	Non Pathological Complete Response (Non PCR, *n* = 11)	0.8 (0.0-2.2)	
CD56^+^	Pathological Complete Response (PCR, *n* = 9)	2.2 (1.0-26.8)	0.010^e^
	Non Pathological Complete Response (Non PCR, *n* = 11)	1.0 (0.0-2.2)	

^(a)^NAC: Neoadjuvant chemotherapy; ^(b)^ ALN: Axillary lymph node; ^(c)^ Average cell count per 400× high-power field (see Materials and Methods); ^(d)^ Mann-Whitney U test; ^e^ Statistically significant

Table 2 documents the median number of cells per HPF found intra-tumourally in metastatic deposits in the tumour-draining ALNs. It shows the predominance of the CD4^+^ and CD8^+^ T cell subsets, the much lower but still prominent level of infiltration by FOXP3^+^ T cells and the low level of infiltration by CTLA-4^+^ T cells and CD56^+^ NK cells (Fig. 2: a, b; c, d; e, f; g, h).

### Higher levels of tumour infiltration by FOXP3^+^ and CTLA-4^+^ T cells, and CD56^+^ NK cells in ALN metastases compared with corresponding primary breast Tumours (*n* = 20): No difference in infiltration by TILs, CD4^+^ and CD8^+^ T cells

The levels of intra-tumoural TILs, CD4^+^ and CD8^+^ T cell subsets in ALN metastatic tumour deposits were comparable with the levels in the corresponding primary breast cancers (Additional file 3: Table S1 and Table 3). There were, however, significantly higher levels of tumour-infiltrating FOXP3^+^ and CTLA-4^+^ T cells in ALN metastases compared with the levels in the corresponding primary breast cancers (*p* = 0.026, *p* = 0.036, respectively). The level of tumour-infiltrating CD56^+^ NK cells was also significantly increased (*p* = 0.006) (Table 3). The CD8^+^: FOXP3^+^ T cell ratio, on the other hand, was not significantly different between the primary breast tumours and the metastatic tumours in the ALNs.

**Table 3:** Comparison of Tumour-infiltrating Lymphocyte Subsets in Primary Breast Tumours and Pre-NAC^(a)^ALN^(b)^ Metastatic Tumours in Women with LLABCs^(c)^

Lymphocyte Subsets (*n* = 20)	Primary Tumours in Breast Median (Range)^(d)^	Metastatic Tumours in ALNs^(b)^ Median (Range)^(d)^	*P* Value^(e)^(Primary Versus Metastases)
CD4^+^	12.8 (0.6-166.2)	26.1 (0.6-157.4)	0.313
CD8^+^	27.4 (0.4-112.6)	37.1 (0.4-160.8)	0.117
FOXP3^+^	5.5 (0.4-96.8)	7.2 (1.0-73.6)	0.026^f^
CTLA-4^+^	0.4 (0.0-2.2)	0.8 (0.0-11.6)	0.036^f^
CD56^+^	0.8 (0.0-3.2)	1.5 (0.0-26.8)	0.006^f^
CD8^+^:FOXP3^+^ ratio	3.91 (0.18-45.00)	3.29 (0.40-21.92)	0.167

^(a)^NAC: Neoadjuvant chemotherapy: ^(b)^ALNs: Axillary lymph nodes (corresponding ipsilateral); ^(c)^LLABCs: Large and locally advanced breast cancers; ^(d)^Average cell count per 400× high-power field (see Materials and Methods); ^(e)^Wilcoxon signed rank test; ^f^Statistically significant

### Positive correlation between tumour-infiltrating lymphocyte subsets (CD8^+^, CD56 ^+^) in primary breast Tumours and metastatic Tumours in ALNs in women with LLABCs

There was a positive correlation between CD8^+^ T and CD56^+^ NK cells infiltrating primary breast cancers and the tumour deposits in metastatic ALNs (rho = 0.514, *p* = 0.020; rho = 0.721, *p* < 0.001, respectively). There was no correlation, however, between CD4^+^, FOXP3^+^ and CTLA-4^+^ T cells infiltrating the primary and metastatic tumours (Additional file 3: Table S2).

### No difference in the lymphocyte profiles (T Effector [CD4^+^, CD8^+^], T regulatory [FOXP3^+^, CTLA-4^+^, PD1^+^] and NK [CD56^+^] cells) in metastatic and non-metastatic ALNs in women with LLABCs

**Fig. 3:**
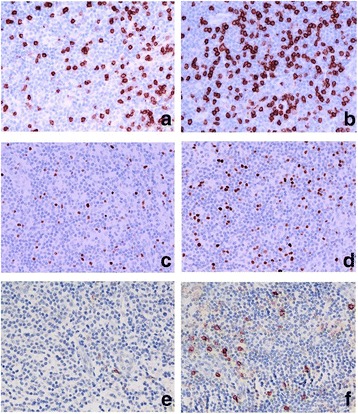
CD8^+^ T cells (**a**, **b**), FOXP3^+^ Tregs (c,dD) and CD56^+^ NK cells (E, F) in the sections of axillary lymph nodes (ALNs), using IHC staining, at 400× magnification. Briefly, heat-mediated antigen retrieval was performed using citrate buffer pH 6 (20 mins). The sections were then incubated with MAbs to CD8 (Dako, M7103) at a 1:100 dilution for 30 mins at RT, MAbs to FOXP3 (Abcam, ab20034) at a concentration of 20 μg/ml for 30 mins at RT, MAbs to CD56 (Dako, M7304) at a 1:50 dilution for 30 mins at RT. Polymeric HRP-linker antibody conjugate was used as secondary antibody. DAB chromogen was used to visualize the staining. The sections were counterstained with haematoxylin. **a**, **c**, **e**: low percentage of CD8^+^ T cells, FOXP3^+^ Tregs and low number of CD56^+^ NK cells respectively; **B**, **d**, **d**: high percentage of CD8^+^ T cells, FOXP3^+^ Tregs and high number of CD56^+^ NK cells respectively. The positively brown membrane-stained cells (CD8^+^ T cells) and brown nuclear-stained cells (FOXP3^+^ Tregs) in non-metastatic paracortical areas of ALNs were quantified as the average % of all cells (5 HPFs). CD56^+^ NK cells were quantified as average number of cell count per HPF in non-metastatic para-cortical areas of ALNs with the greatest accumulation of the positively brown membrane-stained cells

There were no significant differences in the levels (%) of T effector (CD4^+^, CD8^+^), T regulatory (FOXP3^+^, CTLA-4^+^, PD1^+^) and NK (CD56^+^) cells in the tumour-free para-cortical compartments of metastatic ALNs and non-metastatic ALNs (Table 4). Fig. 3 documents CD8^+^ (A, B) and FOXP3^+^ T cells (C, D) and CD56^+^ NK cells in the para-cortical compartment of ALNs.

**Table 4:** Analyses of Lymphocyte Subsets in ALNs^(a)^ in Women with LLABCs^(b)^ Undergoing NAC^(c)^: Comparison of Metastatic and Non Metastatic ALNs

Lymphocyte Subsets (*n* = 33)	Groups	ALN Median (Range)	*P* Value^(g)^
CD4^+^	Non metastatic ALNs (*n* = 9)	63.0 (43.0-74.0)^(e)^	0.796
	Metastatic ALNs (*n* = 24)	68.0 (32.0-75.0)	
CD8^+^	Non metastatic ALNs (*n* = 9)	26.0 (15.4-34.0)^(e)^	0.121
	Metastatic ALNs (*n* = 24)	20.5 (10.4-40.0)	
FOXP3^+^	Non metastatic ALNs (*n* = 9)	4.4 (2.9-8.6)^(e)^	0.736
	Metastatic ALNs (*n* = 24)	4.6 (0.2-10.8)	
CTLA-4^+^	Non metastatic ALNs (*n* = 9)	16.8 (5.2-100.4)^(f)^	0.193
	Metastatic ALNs (*n* = 24)	11.0 (0.6-38.6)	
PD-1^+ (d)^	Non metastatic ALNs (*n* = 9)	6.4 (1.4-36.0)^(f)^	0.408
	Metastatic ALNs (*n* = 7)	12.6 (2.0-72.6)	
CD56^+^	Non metastatic ALNs (*n* = 9)	17.8 (15.8-52.8)^(f)^	0.437
	Metastatic ALNs (*n* = 24)	18.3 (2.2-60.4)	

^(a)^ ALNs: Axillary lymph nodes (paracortical areas: tumour deposits are excluded if present); ^(b)^ LLABCs: Large and locally advanced breast cancers; ^(c)^NAC: Neoadjuvant chemotherapy; ^(d)^ PD-1^+^: Programmed death-1 (*n* = 16); ^(e)^Average percentage of positively stained cells out of all the lymphoid cells in the ALN sections examined; ^(f)^ Average cell count of positively stained cells per 400× high-power field in the ALN sections examined; ^(g)^ Mann-Whitney U test

### High levels of CD8^+^ and low levels of FOXP3^+^ T cell subsets in the Para-cortical compartment (tumour-free) of metastatic ALNs are associated with a PCR following NAC

Comparison between metastatic ALNs with a pCR and without a pCR following NAC demonstrates a significantly high level of CD8^+^ T cells (*p* = 0.048) and low level of FOXP3^+^ T cells (*p* = 0.019) in the para-cortical compartment (tumour-free) of the ALNs. There was no difference in the levels of CD4^+^ and CTLA-4^+^ T cells, nor CD56^+^ NK cells in these ALN response groups (Table 5).

**Table 5:** Analyses of Lymphocyte Subsets in Metastatic ALNs^(a)^ in Women with LLABCs^(b)^ Undergoing NAC^(c)^: Comparison of Metastatic ALNs with a PCR with those without a PCR

Lymphocyte Subsets (*n* = 24)	Groups	ALN Median (Range)	*P* Value^(f)^
CD4^+^	Pathological Complete Response (PCR, *n* = 10)	61.0 (32.0-75.0)^(d)^	0.172
	Non Pathological Complete Response (Non PCR, *n* = 14)	69.0 (36.0-74.0)	
CD8^+^	Pathological Complete Response (PCR, *n* = 10)	27.0 (13.4-40.0)^(d)^	0.048^g^
	Non Pathological Complete Response (Non PCR, *n* = 14)	19.5 (10.4-30.0)	
FOXP3^+^	Pathological Complete Response (PCR, *n* = 10)	3.1 (0.2-6.9)^(d)^	0.019^g^
	Non Pathological Complete Response (Non PCR, *n* = 14)	6.5 (1.7-10.8)	
CTLA-4^+^	Pathological Complete Response (PCR, *n* = 10)	5.7 (0.6-29.6)^(e)^	0.341
	Non Pathological Complete Response (Non PCR, *n* = 14)	11.2 (3.2-38.6)	
CD56^+^	Pathological Complete Response (PCR, *n* = 10)	19.7 (2.2-60.4)^(e)^	0.472
	Non Pathological Complete Response (Non PCR, *n* = 14)	15.9 (6.8-39.0)	

^(a)^ALNs: Axillary lymph nodes (paracortical areas: tumour deposits excluded); ^(b)^LLABCs: Large and locally advanced breast cancers; ^(c)^NAC: Neoadjuvant chemotherapy; ^(d)^Average percentage of positively stained cells out of all the lymphoid cells in the ALN sections (CD4^+^ and CD8^+^ and FOXP3^+^ T cells); ^(e)^Average cell count of positively stained cells per 400× high-power field in the ALN sections (CTLA-4^+^ T cells and CD56^+^ NK cells); ^(f)^Mann-Whitney U test; ^g^ Statistically significant

### High CD8^+^: FOXP3^+^ T cell ratio in ALNs and association with a PCR following NAC

A high CD8^+^: FOXP3^+^ T cell ratio in the para-cortex (tumour-free) of metastatic ALNs was significantly associated with a pCR following NAC. A median of 7.24 was found in ALNs with a pCR versus 3.19 in ALNs without a pCR (*p* = 0.006). Comparison of the CD8^+^: FOXP3^+^ T cell ratios in metastases in ALNs with and without a pCR, however, just failed to reach statistical significance (*p* = 0.080). Moreover, this ratio in corresponding primary tumours was also higher in the pCR group compared with the non-pCR group (7.40 versus 1.48, *p* = 0.002) (Table 6) (data from Kaewkangsadan et al. [13]).

**Table 6:** The Association Between CD8^+^: FOXP3^+^ T Cell Ratio (Breast Tumour, ALN Metastases, ALNs^(a)^ and Subsequent PCR Following NAC^(b)^

Sites	Groups	Median (Range)^(c)^	*P* Value^(d)^ (PCR Versus Non PCR)
Primary breast tumours, *n* = 33 (CD8^+^: FOXP3^+^ T cell ratio)	Tumours with pCR	7.40 (0.27-45.00)	0.002^e^
	Tumours with non pCR	1.48 (0.18-6.04)	
ALN metastatic tumours, *n* = 20 (CD8^+^: FOXP3^+^ T cell ratio)	Metastatic tumours with pCR	5.87 (1.35-21.92)	0.080
	Metastatic tumours with non pCR	1.93 (0.40-7.20)	
ALNs, *n* = 24 (%CD8^+^: %FOXP3^+^ T cell ratio)	ALNs with pCR	7.24 (3.33-75.00)	0.006^e^
	ALNs with non pCR	3.19 (1.78-8.00)	

^(a)^ALNs: Axillary lymph nodes (metastatic but tumour-free paracortical area); ^(b)^NAC: Neoadjuvant chemotherapy; ^(c)^Ratio of CD8^+^:FOXP3^+^ T cells (see Materials and Methods); ^(d)^Mann-Whitney U test; ^e^Statistically significant

### Expression of cytokines (TH1, TH2, TH17, TGF-β) and biological molecules (IDO, PD-L1, VEGF) in ALNs

**Fig. 4:**
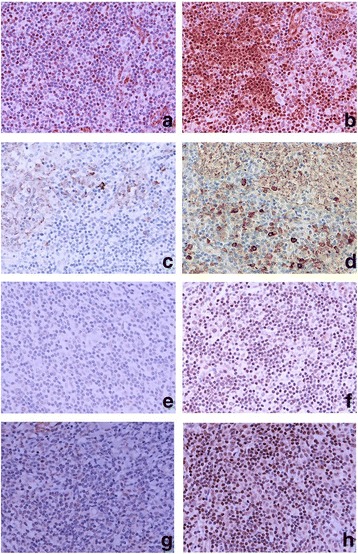
IL-2 (**a**, **b**), IL-10 (**c**, **d**), IL-17 (**e**, **f**) and IFN-γ (**g**, **h**) expression in the sections of axillary lymph nodes (ALNs), using IHC staining, at 400× magnification. Briefly, heat-mediated antigen retrieval was performed using citrate buffer pH 6 (20 mins). The sections were then incubated with MAbs to IL-2 (Abcam, ab92381) at a 1:500 dilution for 30 mins at RT, polyclonal Abs to IL-10 (Abcam, ab34843) at a 1:400 dilution for 30 mins at RT, polyclonal Abs to IL-17 (Abcam, ab9565) at a 1:100 dilution for 30 mins at RT, polyclonal Abs to IFN-γ (Abcam, ab9657) at a concentration of 4 μg/ml for 30 mins at RT. Polymeric HRP-linker antibody conjugate was used as secondary antibody. DAB chromogen was used to visualize the staining. The sections were counterstained with haematoxylin. **a**, **c**, **e**, **g**: low level of expression; **b**, **d**, **f**, **h**: high level of expression. The H score [% of positive cells (brown membrane/cytoplasmic-stained cells) x intensity of staining (1 to 3)] was used to assess the level of expression; low was ≤100 and high was > 100. Scoring performed on non-metastatic areas of a whole ALN section (7-10 HPFs)

A wide range of cytokines and biological molecules were studied in ALNs (metastatic and non-metastatic) (Fig. 4). Significantly higher levels of expression of the Th1 cytokine, IL-2, was found in non-metastatic ALNs (88.9%) compared with metastatic ALNs (14.3%) (*p* = 0.003). A similar profile was seen with the Th1 cytokine, IFN-ϒ (72.8% versus 20%, *p* = 0.049) (Table 7). In contrast, the Th2 cytokine, IL-10, was significantly higher in metastatic compared with non-metastatic ALNs (71.4% versus 22.2%, p = 0.049). There were no significant differences in the levels of expression of transforming growth factor-beta (TGF-β), IL-17, programmed death ligand 1 (PD-L1), indole-amine 2, 3-dioxygenase (IDO) and vascular endothelial growth factor (VEGF) between metastatic and non-metastatic ALNs (Table 7). Thus there was a polarisation from a Th1 to a Th2 profile in tumour-draining metastatic ALNs.

**Table 7:** Expression of Cytokines (Th1, Th2 and Th17), IDO^(a)^, PD-L1^(b)^and VEGF^(c)^ in ALNs^(d)^ in Women with LLABCs^(e)^Undergoing NAC^(f)^

(*n* = 16)	Groups	Low/Negative Expression (*n*)	High Expression (*n*)	Pearson Chi-Square Value	*P* Value
IL-1	Non metastatic ALNs (*n* = 9)	3	6	0.042	0.838
	Metastatic ALNs (*n* = 7)	2	5		
IL-2	Non metastatic ALNs (*n* = 9)	1	8	8.905	0.003^h^
	Metastatic ALNs (*n* = 7)	6	1		
IL-4	Non metastatic ALNs (*n* = 9)	3	6	0.152	0.696
	Metastatic ALNs (*n* = 7)	3	4		
IL-10	Non metastatic ALNs (*n* = 9)	7	2	3.874	0.049^h^
	Metastatic ALNs (*n* = 7)	2	5		
IL-17	Non metastatic ALNs (*n* = 9)	1	8	2.116	0.146
	Metastatic ALNs (*n* = 7)	3	4		
IDO^(g)^	Non metastatic ALNs (*n* = 9)	2	7	2.049	0.152
	Metastatic ALNs (*n* = 7)	4	3		
PD-L1	Non metastatic ALNs (*n* = 9)	7	2	0.085	0.771
	Metastatic ALNs (*n* = 7)	5	2		
IFN-γ	Non metastatic ALNs (*n* = 9)	1	8	3.883	0.049^h^
	Metastatic ALNs (*n* = 7)	4	3		
TGF-β^(g)^	Non metastatic ALNs (*n* = 9)	5	4	0.423	0.515
	Metastatic ALNs (*n* = 7)	5	2		
VEGF	Non metastatic ALNs (*n* = 9)	6	3	0.762	0.383
	Metastatic ALNs (*n* = 7)	6	1		

^(a)^IDO: Indoleamine 2,3-dioxygenase; ^(b)^PDL-1: Programmed death ligand 1; ^(c)^VEGF: Vascular endothelial growth factor; ^(d)^ALNs: Axillary lymph nodes; ^(e)^LLABCs: Large and locally advanced breast cancers; ^(f)^NAC: Neoadjuvant chemotherapy; ^(g)^IDO and TGF-β were scored as negative and positive; ^h^Statistically significant

## Discussion

There is ample evidence that a pCR in the primary breast cancer following NAC is significantly associated with high levels of tumour infiltration by TILs, CD4^+^ and CD8^+^ T effector cells, CD56^+^ NK cells and high CD8^+^: FOXP3^+^ T cell ratios [9, 21, 40, 22, 11, 10, 23, 41, 24–26, 13]. The contribution of the various TIL subsets, however, is inadequately studied and data for several of the subsets is poorly documented.

Droesser et al. [42] found that CD4^+^ T cells infiltrating breast cancer were not a prognostic indicator. Heys et al. [43] reported low levels of CD4^+^ T cells to be significantly associated with a better response to NAC. In contrast, we documented that high levels of CD4^+^ T cells, intratumoural and stromal, in LLABCs were significantly associated with a pCR following NAC [13]. Mahmoud et al. [44] described that high levels of CD8^+^ T cells were independently associated with longer breast cancer-specific survival. Matkowski et al. [45], however, showed that a high level of CD8^+^ T cells in specific types of breast cancers (high tumour grade, metastatic spread to ALNs) was associated with a poor prognosis. A small number of studies, including our own, have found that high levels of CD8^+^ T cells in primary breast cancers were associated with a pCR following NAC [22, 13]; a high CD8^+^: FOXP3^+^ T cell ratio was also significantly associated with a pCR [21, 13].

The role of TILs, T effector (CD8^+^, CD4^+^) and T regulatory (FOXP3^+^, CTLA-4^+^, PD-1^+^) cells and CD56^+^ NK cells in ALNs is even less well studied and their contribution to the induction of immune-mediated tumour cell death in ALN metastases poorly documented. A small number of studies have been carried out in sentinel lymph nodes (SLNs) and non SLNs from the axilla in women with breast cancer. SLNs are the first group of lymph nodes draining the primary tumour in the breast and are thus the first immune barrier to disseminating cancer cells [31–33].

Korht et al. [31] showed that increased levels of CD4^+^ and CD8^+^ T effector cells in both SLNs and ALNs correlated with an improved 5 year DFS. The ALN but not the SLN immune profile, on the other hand, was independent of the presence of metastatic disease in ALNs [31]. Mansfield et al. [33] documented enhanced CD8^+^ T cell levels in SLNs, with or without metastases. We did not perform SLN biopsies in the women undergoing surgery post-NAC in our study group. Our study showed no differences in the CD4^+^ and CD8^+^ T cell subsets between metastatic (tumour-free areas) and non-metastatic ALNs and is in agreement with the findings described above.

CD4^+^ T cells consist of different T helper cells (Th1, Th2, Th17), secreting a wide range of pro- and anti-inflammatory cytokines, as well as producing natural and inducible CD4^+^CD25^+^FOXP3^+^ Tregs, and show a degree of plasticity in terms of function [46]. CD8^+^ T cells also consist of different subsets - naïve, memory and activated CD8^+^ cytotoxic T lymphocytes (CTLs) and weak suppressor cells [47]. It was not possible to attribute precisely the contribution of the different CD4+ /CD8+ subsets to the pCR with NAC.

We have documented recently the important contribution of CD56^+^ NK cells to a pCR with NAC in LLABCs. High levels of CD56^+^ NK cell concentration in the primary tumour, intra-tumoural or stromal, were associated with good pathological responses and pCRs, and shown to be an independent predictor for a pCR [26]. In the current study, high levels of CD56^+^ NK cells infiltrating metastatic deposits in ALNs were found to be similarly significantly associated with pCRs following NAC. Interestingly, there was no difference in the CD56^+^NK cell subset present in the para-cortical compartment of metastatic (tumour-free areas) and non-metastatic ALNs. To the best of our knowledge, these findings in ALNs in human breast cancer have not previously been described.

CD56^+^ NK cells have been shown to play an important role in tumour immune surveillance, in the prevention of progressive tumour growth and in the defence against metastatic dissemination [26]. Most human solid tumours have low levels of infiltration by CD56^+^ NK cells. A prominent infiltration, however, is usually associated with an improved prognosis and reduction of tumour recurrence [48–51]. Our results in the current study are in agreement with these published findings, as a pCR in tumour and lymph nodes in breast cancer is a surrogate marker of a good clinical outcome [27, 28].

T regulatory cells are generated during the immune response and suppress the function of a wide range of immune cells (T effector [CD4^+^, CD8^+^], NK and DCs) [52, 53]. Blood and tumour-infiltrating Tregs (FOXP3^+^, CTLA-4^+^, PD-1^+^) play a crucial role in controlling the anti-cancer cellular immune responses in the circulation and tumour microenvironment [54, 13]. High levels of FOXP3^+^ T cells have been reported infiltrating invasive breast cancers and to be significantly increased in both HER2 positive and triple-negative breast cancers [55–59, 13].

Oda et al. [22] documented that high levels of FOXP3^+^ T cells in the primary tumour prior to NAC were associated with high pCR rates. Moreover, Demir et al. [38] stated that high levels of FOXP3^+^ T cell infiltration post-NAC correlated with enhanced rates of pCR. In contrast, we have shown that NAC reduced both blood and tumour FOXP3^+^ T cells concurrently in patients with LLABCs and that high levels of FOXP3^+^ T cells in blood and tumour following NAC were associated with a poor pathological response [13]. In breast cancer, NAC has been well documented to significantly reduce tumour- infiltrating FOXP3^+^, CTLA-4^+^ and PD-1^+^ T cells (but not CD8^+^ T cells) [60, 61, 38, 25, 13].

The profile and the function of FOXP3^+^ T cells in tumour-draining ALNs is less well studied. FOXP3^+^ T cells have been shown to be increased in numbers in SLNs, in particular in metastatic nodes; even micro-metastatic disease was associated with increased levels of FOXP3^+^ T cells [62, 63, 32, 33]. In our study, high levels of FOXP3^+^ and CTLA-4^+^ T cells were documented in metastatic tumours in the ALNs and were higher than the levels in the corresponding primary tumours. A low % of FOXP3^+^ T cells (and high % of CD8^+^ T cells) in para-cortical (tumour-free) areas of metastatic ALNs was significantly associated with ALN pCRs. Such findings have not been documented in the literature.

CTLA-4 is a co-inhibitory receptor molecule found on activated and exhausted T cells and Tregs and negatively regulates T cell interaction with CD80/CD86 ligand binding sites [64, 65]. In primary breast cancers there is an increased expression of CTLA-4, compared with normal breast tissue [66]. High levels of CTLA-4 mRNA in primary breast cancers were shown to be associated with ALN metastases and advanced tumours [66, 67]. We have previously demonstrated high levels of CTLA-4^+^ T cells in the blood of women with LLABCs [54]. Although high levels of tumour-infiltrating FOXP3^+^ T cells (and PD-1^+^ lymphocytes) were not associated with a pCR following NAC, tumour stromal infiltration by high levels of CTLA-4^+^ T cells were. The in situ CTLA-4^+^ expression was likely to be due to activated T cells [13]. In our study in ALNs, higher levels of CTLA-4^+^ T cells were demonstrated in ALN metastases than in the corresponding primary tumours. In contrast to the findings in the primary breast cancers, high levels of CTLA-4^+^ T cells in ALNs were not significantly associated with a pCR following NAC. There is, however, a dearth of publications regarding CTLA-4^+^ T cells and breast cancer, either in the primary tumour or ALNs.

PD-1 is expressed on activated and exhausted T cells, Tregs, NK cells and DCs [68, 16, 69]. On interacting with PD-L1/L2 in a co-inhibitory pathway in tissues it down-regulates activated T cells resulting in T cell tolerance and prevention of auto-immunity [70]. The PD-1/PD-L1 pathway is a key immune check-point exploited by malignant cells to escape anti-cancer immune defences [71].

High levels of PD-1^+^ lymphocytes have been shown to have a significant correlation with reduced patient survival [72]. In our primary breast cancer study the levels of PD-1^+^ cells were low and there was no association with a subsequent pCR following NAC [13]. Comparable findings were documented in ALN metastases. PD-1^+^ T cell subsets have not previously been described in ALNs in breast cancer; we found no difference in the T regulatory profiles between metastatic and non-metastatic ALNs.

In various human cancers malignant cells and host infiltrating cells express and secrete a range of Th1, Th2 and Th17 cytokines and TGF-β which enhance or suppress the in situ anti-cancer immune responses [73–77]. In such studies the semi-quantitative methods used did not discriminate between the tumour-infiltrating immune and malignant cells, nor quantify the contribution of the different cells to the cytokine profiles in the tumour [13].

We have previously demonstrated a polarisation of Th2 production in vitro by blood lymphocytes from women with LLABCs; this polarisation persisted post-NAC [54]. Our current study has also revealed the presence of a Th2 cytokine polarisation in ALNs with metastases. There was a high level of expression of the Th2 suppressive cytokine IL-10 and low level of expression of the Th1 inflammatory cytokines INF-ϒ and IL-2, when compared with non-metastatic ALNs. Interestingly, Matsuura et al. [32] noted in breast cancer that micro-metastases in SLNs stimulated Th1 responses (T-box family of transcription factors) whilst macro-metastases enhanced Th2 responses (GATA family of zinc finger proteins). This area of immune reactivity in ALNs is poorly studied.

The role of IL-17 in human cancer is not well defined. In a study in breast cancer, the level of Th17 cells was shown to be increased and associated with a good clinical outcome [76]. In human cancers, TGF-β expression is usually upregulated. It induces the production of FOXP3^+^ Tregs, inhibiting the generation and activity of innate and adaptive immunity [53, 78]. High levels of expression of IL-10 and IL-17 in breast cancer following NAC have been shown to be significantly associated with failure to achieve a pCR [13]. In our current study with ALNs we did not demonstrate any significant changes in expression of IL-17 and TGF-β, as well as PD-L1, VGF and IDO, between tumour-free and metastatic ALNs.

Although most chemotherapeutic agents inhibit elements of innate and adaptive immunity, they can enhance both components, resulting in immune-mediated tumour cell death [79, 17, 20]. Chemotherapy induces cancer cell stress and damage which results in the release of “danger signals” and immunogenic tumour-associated antigens (TAAs). Danger signals activate innate immune cells whilst TAAs are taken up by DCs resulting in the release of pro-inflammatory cytokines and the production of anti-cancer CTLs. Anthracyclines, in particular, induce tumour cell damage and release/expose calreticulin and other endoplasmic proteins [79, 80, 19]. The NAC combination used in our trial consisted of anthracycline, cyclophosphamide, taxane, ± capecitabine. These chemotherapeutic agents are known to have immunomodulatory effects. Doxorubicin (anthracycline) can enhance the production of TAA-specific CD8^+^ CTLs and induce tumour infiltration by CD8^+^ T cells [16, 81]. Cyclophosphamide inhibits the generation and function of FOXP3^+^ Tregs [15, 82]. Docetaxel (taxane) has been shown to increase serum IFN-ϒ, IL-2 and IL-6 levels and NK cell activity in blood [83, 14]. Capecitabine is enzymatically converted to 5-fluorouracil (5-FU) and this enhances the expression of TAAs on tumour cells and antibody-dependent cell-mediated cytotoxicity [84, 85]. Thus NAC induces a range of anti-cancer immune responses which contribute to the damage and eradication of malignant cells.

Our current and previous findings suggest that the immune milieu in the breast and ALNs in patients with LLABCs plays a key role in inducing tumour cell death, both in the primary cancer and ALN metastases in patients undergoing NAC. High levels of TILs, CD4^+^ and CD8^+^ T cells and NK cells in the primary and ALN metastases were associated with significant pCRs. There was no alteration in levels of infiltration by TILs and a positive correlation between CD8^+^ T and NK cells infiltrating primary and metastatic tumours [26]. To the best of our knowledge, there are no publications regarding TILs, Tregs (FOXP3^+^, CTLA-4^+^) and NK cells in metastatic tumours in ALNs, nor comparisons with corresponding primary breast cancers. In our NAC trial in LLABCs concurrent pCRs at both sites was infrequent but were associated with the best clinical outcome; a less beneficial outcome was seen with a pCR in the breast alone [28].

## Conclusions

Our study of tumour-draining ALNs in women with LLABCs undergoing NAC has demonstrated new and important findings, complementing the results previously documented in the primary tumours. We have characterised further the key contributions of tumour-infiltrating TILs, T effector (CD4^+^, CD8^+^), T regulatory (FOXP3^+^, CTLA-4^+^) and CD56^+^ NK cells to pCRs in ALN metastases. High levels of CD8^+^ T cells and low levels of FOXP3^+^ T cells in para-cortical areas (tumour-free) were associated with pCRs following NAC. Th2 polarisation (high IL-10, low IL-2 and IFN-ϒ) was present in ALNs with metastases. In LLABCs, the close interrelationship between a pCR in breast and ALNs and the concomitant immune changes induced by NAC suggests that immune-mediated cell death may be a crucial component of NAC-associated tumour cell destruction and removal.

## Additional files

Additional file 1: Table S3. Patient and Tumour Characteristics, Responses to Neoadjuvant Chemotherapy (*n* = 33). (DOCX 56 kb)

Additional file 2: Table of Patient Characteristics. (DOCX 15 kb)

Additional file 3: Table S1. Comparison of Tumour-infiltrating Lymphocytes (TILs) in Primary Breast Tumours and Pre-NAC^(1)^ ALN^(2)^ Metastatic Tumours in Women with LLABCs^(3)^. There was no significant difference between the levels of TILs in primary breast tumours and axillary metastatic tumour deposits. **Table S2. **Correlations of Tumour-infiltrating Lymphocyte Subsets in Primary Breast Tumours and ALN ^(1)^ Metastatic Tumours in Women with LLABCs^(2)^ [Spearman’s Correlation Coefficient (rho)] (*n* = 20). There was a positive correlation between CD8^+^ T and CD56^+^ NK cells infiltrating primary breast cancers and the tumour deposits in metastatic ALNs (rho=0.514, *p* =0.020; rho=0.721, *p* < 0.001, respectively). There was no correlation, however, between CD4^+^, FOXP3+ and CTLA-4^+^ T cells infiltrating the primary and metastatic tumours. (DOCX 26 kb)

## Abbreviations

5-FU: 5-fluorouracil
A: Adriamycin
ALN: Axillary lymph node
C: Cyclophosphamide
CD: Cluster of differentiation
CTL: Cytotoxic T lymphocyte
CTLA-4: Cytotoxic T lymphocyte antigen 4
DAB: Di-amino-benzidine
DC: Dendritic cell
DFS: Disease-free survival
ER: Oestrogen receptor
FOXP3: Forkhead box protein 3
H&E: Haematoxylin and eosin
HER2: Human epidermal growth factor receptor 2
HPF: High-power field
HRP: Horseradish peroxidase
IFN-γ: Interferon-gamma
IHC: Immunohistochemistry
IL: Interleukin
LLABC: Large locally advanced breast cancer
MAb: Monoclonal antibody
NAC: Neoadjuvant chemotherapy
NK: Natural killer
OS: Overall survival
pCR: Pathological complete response
PD-1: Programmed death 1
PD-L1: Programmed death ligand 1
RT: Room temperature
SLN: Sentinel lymph node
T: Docetaxel
TAA: Tumour-associated antigen
TGF-β: Transforming growth factor-beta
Th: T helper
TIL: Tumour-infiltrating lymphocyte
Treg: T regulatory cell
X: Capecitabine
