# Supplementary material for: Tumour-draining axillary lymph nodes in patients with large and locally advanced breast cancers undergoing neoadjuvant chemotherapy (NAC): the crucial contribution of immune cells (effector, regulatory) and cytokines (Th1, Th2) to immune-mediated tumour cell death induced by NAC
Source: BMC Cancer. 2018 Feb 2;18:123. doi: 10.1186/s12885-018-4044-z (PMC5795830; doi:10.1186/s12885-018-4044-z)
Supplement: Supplementary file 1 — Patient and Tumour Characteristics, Responses to Neoadjuvant Chemotherapy (n = 33). (DOCX 56 kb) [file 12885_2018_4044_MOESM1_ESM.docx]

**Table S3.** Patient and Tumour Characteristics, Responses to Neoadjuvant Chemotherapy (n=33)

^(1)^ BMI: Body mass index (≤30: Non-obese, >30: Obese)

^(2)^ Menopausal status: Pre-menopausal, age < 55 years with normal menstrual cycles; Post-menopausal, age > 50 years with no spontaneous menses for at least one year/ or age ≤ 50 years with no spontaneous menses within the past 2 years/or women who had bilateral oophorectomy prior to the diagnosis of breast cancer

^(3)^ Histological grade: Grade 1 (well differentiated), grade 2 (moderately differentiated), grade 3 (poorly differentiated)

(4) ER (oestrogen receptor): Allred scoring system was used for measuring expression of ER (score ≥ 3 for positive, < 3 for negative)

^(5)^ HER2 (human epidermal growth factor receptor 2): Determined by FISH (fluorescence in-situ hybridisation)

^(6)^ A: Adriamycin (doxorubicin), C: Cyclophosphamide, T: Taxotere (docetaxel) and X: Xeloda® (capecitabine)

^(7)^ Clinical response was assessed by MRI (magnetic resonance imaging) of breast after 2 cycles of AC using the RECIST criteria

^(8)^ Pathological response in breast was graded as grade 1: No change or some alteration to individual malignant cells but no reduction in overall cellularity; grade 2: A minor loss of tumour cells but overall cellularity still high, up to 30 % loss; grade 3: Between an estimated 30% and 90% reduction in tumour cells; grade 4: A marked disappearance of tumour cells such that only small clusters or widely dispersed individual cells remain, more than 90% loss of tumour cells; grade 5: No malignant cells identifiable from the site of the tumour (pCR)

^(9)^ Pathological response in axilla was graded as grade 1: Metastasis with no fibrosis; grade 2: Metastasis with variable replacement by fibrous tissue; grade 3: No malignant cells identifiable but replacement by fibrous tissue (pCR); NA (not applicable): No nodal metastasis

^(10)^ Recurrent disease and death from a median follow-up of 51 months
