# Supplementary material for: Tumour-draining axillary lymph nodes in patients with large and locally advanced breast cancers undergoing neoadjuvant chemotherapy (NAC): the crucial contribution of immune cells (effector, regulatory) and cytokines (Th1, Th2) to immune-mediated tumour cell death induced by NAC
Source: BMC Cancer. 2018 Feb 2;18:123. doi: 10.1186/s12885-018-4044-z (PMC5795830; doi:10.1186/s12885-018-4044-z)
Supplement: Supplementary file 2 — Table of Patient Characteristics. (DOCX 15 kb) [file 12885_2018_4044_MOESM2_ESM.docx]

| **Table of Patient Characteristics (N=33)** | |
| --- | --- |
| **Groups** | **N** |
|  |  |
| Age (years) |  |
| <50 | 14 |
| ≥50 | 19 |
|  |  |
| Menopausal |  |
| pre | 16 |
| post | 17 |
|  |  |
| Tumour size |  |
| <40 mm | 18 |
| ≥40 mm | 15 |
|  |  |
| Nodal status |  |
| negative | 9 |
| positive | 24 |
|  |  |
| Tumour grade |  |
| 1(low) | 2 |
| 2(moderate) | 13 |
| 3(high) | 18 |
|  |  |
| ER^(1)^ status |  |
| negative | 11 |
| positive | 22 |
|  |  |
| HER-2 status |  |
| negative | 23 |
| positive | 10 |
|  |  |
| NAC regimen |  |
| AC-TX^(2)^ | 16 |
| AC-T | 17 |
|  |  |
| ^(1)^ ER: Oestrogen receptor; ^(2)^ AC-TX: Doxorubicin, cyclophosphamide, taxotere and Xeloda® (capecitabine), respectively | |
