# Supplementary material for: Tumour-draining axillary lymph nodes in patients with large and locally advanced breast cancers undergoing neoadjuvant chemotherapy (NAC): the crucial contribution of immune cells (effector, regulatory) and cytokines (Th1, Th2) to immune-mediated tumour cell death induced by NAC
Source: BMC Cancer. 2018 Feb 2;18:123. doi: 10.1186/s12885-018-4044-z (PMC5795830; doi:10.1186/s12885-018-4044-z)
Supplement: Supplementary file 3 — Comparison of Tumour-infiltrating Lymphocytes (TILs) in Primary Breast Tumours and Pre-NAC(1) ALN(2) Metastatic Tumours in Women with LLABCs(3). There was no significant difference between the levels of TILs in primary breast tumours and axillary metastatic tumour deposits. Table S2. Correlations of Tumour-infiltrating Lymphocyte Subsets in Primary Breast Tumours and ALN (1) Metastatic Tumours in Women with LLABCs(2) [Spearman’s Correlation Coefficient (rho)] (n = 20). There was a positive correlation between CD8+ T and CD56+ NK cells infiltrating primary breast cancers and the tumour deposits in metastatic ALNs (rho=0.514, p =0.020; rho=0.721, p < 0.001, respectively). There was no correlation, however, between CD4+, FOXP3+ and CTLA-4+ T cells infiltrating the primary and metastatic tumours. (DOCX 26 kb) [file 12885_2018_4044_MOESM3_ESM.docx]

| **Table S1 Comparison of Tumour-infiltrating Lymphocytes (TILs) in Primary Breast Tumours and Pre-NAC^(1)^ ALN^(2)^ Metastatic Tumours in Women with LLABCs^(3)^** | | | | | |
| --- | --- | --- | --- | --- | --- |
| **Groups** | | | **Metastatic Tumours in ALNs** | | **P Value^(4)^ (Primary Versus Metastases)** |
|  |  |  | **Low Infiltration (n)** | **High Infiltration (n)** |  |
| TILs | Primary Tumours in Breast | Low Infiltration (n) | 11 | 2 | 1.000 |
|  |  | High Infiltration (n) | 3 | 4 |  |
|  |  |  |  |  |  |
| ^(1)^ NAC: Neoadjuvant chemotherapy; ^(2)^ ALNs: Axillary lymph nodes (corresponding ipsilateral); ^(3)^ LLABCs: Large and locally advanced breast cancers; ^(4)^ Related-Samples McNemar Test | | | | | |

| **Table S2 Correlations of Tumour-infiltrating Lymphocyte Subsets in Primary Breast Tumours and ALN ^(1)^ Metastatic Tumours in Women with LLABCs^(2)^ [Spearman's Correlation Coefficient (rho)] (n=20)** | | | | | | |
| --- | --- | --- | --- | --- | --- | --- |
| **Lymphocyte Subsets** | | **Metastatic Tumours in ALNs** | | | | |
|  |  | **CD4^+^** | **CD8^+^** | **FOXP3^+^** | **CTLA-4^+^** | **CD56^+^** |
| **Primary Tumours in Breast** | **CD4^+^** |  |  |  |  |  |
|  | Correlation Coefficient | 0.061 | NA^(3)^ | NA | NA | NA |
|  | P Value (2-tailed) | 0.797 | NA | NA | NA | NA |
|  | **CD8^+^** |  |  |  |  |  |
|  | Correlation Coefficient | NA | 0.514 | NA | NA | NA |
|  | P Value (2-tailed) | NA | 0.020* | NA | NA | NA |
|  | **FOXP3^+^** |  |  |  |  |  |
|  | Correlation Coefficient | NA | NA | 0.390 | NA | NA |
|  | P Value (2-tailed) | NA | NA | 0.089 | NA | NA |
|  | **CTLA-4^+^** |  |  |  |  |  |
|  | Correlation Coefficient | NA | NA | NA | 0.246 | NA |
|  | P Value (2-tailed) | NA | NA | NA | 0.296 | NA |
|  | **CD56^+^** |  |  |  |  |  |
|  | Correlation Coefficient | NA | NA | NA | NA | 0.721 |
|  | P Value (2-tailed) | NA | NA | NA | NA | <0.001* |
|  |  |  |  |  |  |  |
| ^(1)^ ALN: Axillary lymph node; ^(2)^ LLABCs: Large and locally advanced breast cancers; ^(3)^ NA: Not applicable; * Statistically significant | | | | | | |
